# Clinical development innovation in rare diseases: lessons learned and best practices from the DevelopAKUre consortium

**DOI:** 10.1186/s13023-021-02137-0

**Published:** 2021-12-14

**Authors:** Mattias Rudebeck, Ciarán Scott, Nicholas P. Rhodes, Christa van Kan, Birgitta Olsson, Mohammed Al-sbou, Anthony K. Hall, Nicolas Sireau, Lakshminarayan R. Ranganath

**Affiliations:** 1 Swedish Orphan Biovitrum (Sobi), Stockholm, Sweden; 2 AKU Society, Cambridge, CB1 2BL UK; 3 Liverpool University Hospitals NHS Foundation Trust, Liverpool, UK; 4 PSR Group, Hoofddorp, The Netherlands; 5 Faculty of Medicine, Mutah University, Karak, Jordan; 6 Cudos, Hoofddorp, The Netherlands

**Keywords:** Rare diseases, Drug development, Alkaptonuria, AKU, DevelopAKUre, Best practice

## Abstract

New opportunities have arisen for development of therapies for rare diseases with the increased focus and progress in the field. However, standardised framework integrating individual initiatives has not been formed. We present lessons learned and best practice from a collaborative success case in developing a treatment for a rare genetic disease. Our unique consortium model incorporated several of the identified developments under one project, DevelopAKUre, truly bringing together academia, industry and patient organisations in clinical drug development. We found that the equal partnership between all parties in our consortium was a key success factor creating a momentum based on a strong organisational culture where all partners had high engagement and taking ownership of the entire programme. With an agreed mutual objective, this provided synergies through connecting the strengths of the individual parties. Another key success factor was the central role of the patient organisation within the management team, and their unique study participants’ advocacy role securing the understanding and meeting the needs of the clinical study participants in real-time. This resulted in an accelerated enrolment into the clinical studies with a high retention rate allowing for delivery of the programme with significantly improved timelines. Our project was partly funded through an external EU research grant, enabling our model with equal partnership. Further attention within the community should be given to establishing a functional framework where sustainable funding and risk sharing between private and public organisations allow for our model to be replicated.

## Background

Developing new treatments for medical conditions is a complex field of medicine with a significant number of challenges, particularly for rare diseases. The success rates of clinical development programmes have been found to be low with only 6.2% (13.2% when excluding oncology indications) of orphan drug developmental candidates reaching the market, with success rates for both Phase 2 and 3 being less than 50% [1].

Of the estimated 7000 rare diseases that affect more than 400 million people globally, around 95% currently have no approved treatment [2]. There are many reasons for this, some for which actions have been taken to promote a higher level of effort, such as those of regulatory agencies to stimulate pharmaceutical industry investments [3]. However, some of the core reasons include the lack of understanding of the biology of rare diseases, navigating regulatory pathways for these, and defining and developing outcome measures relevant for the specific rare disease [4]. Further, major challenges relate to designing and running clinical studies in extremely small patient populations. The low prevalence of an often heterogenous group, provides a small pool of known patients, which reduces the potential to recruit the required number of participants, but also creates logistical issues with patients being spread across countries and continents with long distances to the nearest treatment centre. Even for clinical studies in more common diseases, recruitment of study participants is a major challenge. The proportion of the clinical development time spent on recruitment has been found to be up to 30% [5], and 86% of studies are delayed due to patient recruitment issues, with data suggesting an extension of recruitment timelines by a factor of two compared to initial plan [6].

Once the study recruitment has been completed, the retention of study participants has also proved to be a major challenge in the conduct of clinical studies. Here, data show that 54% and 53% of study participants report that being provided with supporting information on the clinical research or information on managing their health condition influenced their participation in clinical studies, [7]. Sixty-one percent reported that feeling part of a community was a major reason for them to remain in a study [8]. It is important to incorporate an understanding of participants´ experiences in clinical studies in the recruitment and retention planning as these issues increase both costs and timelines and, more importantly, also have an impact on the lives of the people affected by the disease [4].

In recent decades, a focus on rare diseases has resulted in an increased awareness of the community and specific diseases, which has progressed the field. This can be seen in media visibility and overall attention, including funding of rare disease research and targeted drug development by governments. The development of specialised centres caring for rare disease patients within academic medical institutions has further contributed to this change. A major factor to the advancement of the field would also be the professional development of rare disease advocacy groups. An increased number of patient organisations have emerged since the technical revolution further enabled online communities to connect individual patients with each other. This has led to patient advocacy groups becoming larger and with greater influence [9]. This has in turn led to opportunities for further partnerships between these groups and the pharmaceutical industry which provides crucial input and collaboration on clinical development, especially within rare diseases.

At the same time, partnerships between academic institutions, health care providers and the pharmaceutical industry provide further opportunities to integrate diverse key skills into a collaboration for successful planning and execution of clinical research. The industry partner provides the skills of delivering international, multicentre and complex clinical studies meeting regulatory requirements, as well as resources needed to perform such research programmes, manufacturing the pharmaceutical product and ensuring that a positive clinical programme outcome is brought to regulatory assessment and subsequently with ensured patient access. The academic partner provides high medical and scientific competence input to the clinical development programme as well as the research equipment needed for scientifically robust planning and execution [9].

Taking all the above aspects together, new opportunities have arisen for development of therapies for rare diseases. However, despite implementation of several initiatives, a standardised framework integrating these elements has not been established [10] which hinders a radical paradigm shift.

Herein we present lessons learned and best practice from a European collaborative success case in developing a treatment for the rare genetic disease alkaptonuria (AKU). Our unique consortium model incorporated several of the identified developments above under one project. In 2020, the outcome of our research led to the regulatory approval of the first pharmacological treatment for AKU [11].

### Specific research methodological challenges in clinical development in AKU

First of all, it is important to understand the specific methodological challenges in performing research in AKU due to the characteristics of the disease. AKU is a rare, serious, autosomal recessive multisystem disorder [12] affecting approximately one in every 250 000 to 1 000 000 people [13].

AKU is caused by a genetic deficiency of an enzyme, homogentisate 1,2-dioxygenase, involved in tyrosine metabolism. This results in accumulation of homogentisic acid (HGA) which is then progressively converted to a melanin-like HGA-pigment which deposits in connective tissues, causing them to become rigid and eventually brittle and prone to degradation. This disease-specific process is called ochronosis [14, 15], characterised by multisystem involvement with variable phenotypes, comprising severe premature spondyloarthritis, lithiasis, cardiac valve disease, fractures, muscle and tendon ruptures, and osteopenia [16, 17]. The disease is slowly progressing with a pre-symptomatic phase, other than dark urine, until clinical signs and symptoms appear, usually when patients are in their late 20 s [18]. For our research, the multifaceted aspects of the disease required the correct endpoints to be defined as well as large amounts of data to be collected, meaning complex studies to perform and for patients to participate in. At the same time, these aspects also require a large sample size for statistical power of the study. These factors provided our programme with significant challenges related to patient recruitment, which will be covered specifically in this paper.

Alkaptonuria is a disorder of tyrosine metabolism similar to hereditary tyrosinaemia type 1 (HT-1) for which nitisinone has been approved since 2002 in the United States and since 2005 in Europe [19]. Nitisinone is an inhibitor of the enzyme 4-hydroxyphenylpyruvate dioxygenase, further upstream from the defects in AKU and HT-1, which thereby prevents formation of toxic or harmful metabolites. An initial clinical study with nitisinone in AKU was however inconclusive for the efficacy analyses [20], learnings which we took into consideration in the planning of our studies, resulting in a large amount of data for all aspects of the disease being collected and analysed. As nitisinone was proven to lower HGA levels, thus preventing the darkening of urine in patients with AKU, masking is not possible, an aspect providing challenges with meeting regulatory authority requirements of blinded, randomised controlled studies. We therefore designed an open study where the untreated control group did not receive a placebo. Instead, the study was evaluator-blinded as far as possible [21]. Another challenge was that clinical studies in AKU require a long study period (we used 4 years for our Phase 3 study) due to the slow progression of the disease. The risk of dropouts over the study years, which could introduce bias in the statistical analysis, was considered high, especially in the untreated control group. An important aspect was therefore to ensure high patient retention, which will also be covered specifically in this paper.

## Identified success factors

Our team has identified the main factors that contributed to us being able to deliver the clinical development programme with extraordinary operational excellence. These will be presented in the coming sections.

### Preparation—key to success

Before the start of our clinical development programme for AKU, the understanding of the disease was still rudimentary. A scientific plan was executed to prepare for the clinical research phase. Knowledge of the impact of the disease on the human body was investigated by autopsy of a patient with AKU, enabled by post-mortem donation [17]. By development of an in vitro model, the link between the culprit disease molecule and the disease outcome was demonstrated pre-clinically [22] as well as the development of an animal model to investigate the potential pharmacological treatment [23]. The natural history of the disease was further established [24] and a clinical evaluation tool to assess disease progression in this multisystem and slowly progressing disease was developed [25], both important for planning the study design. Furthermore, an early interaction with the regulatory agency (EMA, given the initial programme focus on Europe) to discuss the study programme gave important input which improved the design of the Phase 3 study.

#### Key considerations: Preparation

- Ensure execution of scientific plan to gain sufficient understanding of the rare disease, including natural history, prior to design of clinical programme
- Ensure valid clinical evaluation tools are available or developed
- Ensure understanding of the clinical relevance and patient perspective of endpoints
- Ensure early interaction with regulators

In parallel with the planning of the clinical development programme, the important disease and patient community network was created, allowing for the successful conduct and completion of the programme.

### Building a consortium

The initiating patient organisation (AKU Society, United Kingdom) together with the leading clinical experts (Royal Liverpool University Hospital, United Kingdom (consortium coordinator)) and academic researchers (University of Liverpool, United Kingdom) identified the skills required to successfully conduct an international multicentre clinical development programme that would meet the regulatory requirements. In total, 12 partner organisations from academia, healthcare, industry, and patient advocates were recruited to a consortium founded in 2012 that was named DevelopAKUre (pronounced ‘develop a cure’). These are described in Table 1.

**Table 1:** Identified skills required and members of DevelopAKUre consortium

Expertise	Partner
Leading clinical disease expertise and coordination	Royal Liverpool University Hospital (RLUH) (consortium coordinator, study co-sponsor)
Leading scientific disease expertise	University of Liverpool (study co-sponsor)
Patient perspective and network, advocacy	AKU Society, United Kingdom
Operational clinical study expertise (including Data Management) to conduct complex international clinical studies in rare diseases	PSR, the Netherlands
Clinical research from a regulatory perspective, managing regulatory authority interactions and applications, expertise in the investigational drug and pharmacovigilance	Swedish Orphan Biovitrum (Sobi), Sweden
Medical monitoring expertise	Cudos, the Netherlands
Biomarker analysis expertise	Nordic Biosciences, Denmark
Inflammatory markers analysis expertise	Universita degli studi di Siena, Italy
Genetic analysis expertise	Biomedical Research Center of the Slovak Academy of Sciences, Slovakia
Clinical expertise and study conduct (in addition to RLUH)	Hôpital Necker, France (study site)
Clinical expertise and study conduct (in addition to RLUH)	National Institute of Rheumatic Diseases, Slovakia (study site)
Additional clinical study participants’ support and communication with study participants	ALCAP, France

The AKU Society continued to support establishment of sister organisations globally which became part of the international network that enabled the programme to be executed and well managed.

A strong leadership of the consortium enabled efficient communication and timely issue solving. The consortium held weekly project board virtual meetings with mandatory representation from all partners ensuring coordination and progress of all deliverables, and all issues arising during the programme could immediately be solved or followed up.

The consortium was able to obtain external funding through the European Commission’s 7^th^ Framework Programme (FP7; project number 304985) that partly covered the costs of the clinical development programme. This enabled equal partnership within the consortium for all member organisations, where one partner was not the funding sponsor which would not have allowed for the equality that nurtured ownership and engagement by all partners.

The parties established and agreed on a detailed consortium contract prior to the commencement of the work. This legal document specified the relationship between the parties, in particular concerning the organisation of work amongst the parties, the management of the project and the rights and obligations of the parties as well as dispute resolution.

The coordination and leadership in combination with the high engagement and ownership by each consortium partner resulted in the extraordinary timelines demonstrated in this project.

#### Key considerations: Building a consortium

- Identify skills and talent and establish a consortium with complementary disciplines
- Ensure timely cross-functional communication by weekly mandatory meetings
- Ensure ownership and engagement of all parties for the full project by equal partnership
- Ensure a binding contract is agreed prior to commencement and cover all aspects of the project’s lifecycle

### Funding

Funding for rare diseases is difficult. The fact that the disease is rare means that there are few funding opportunities. When funding opportunities are available, researchers should consider strategic use of experts with clear knowledge and experience of the funder’s procedures and ensure that the research programme satisfies the core purpose of the funding. Pan-national organisations such as the European Commission, need to demonstrate that any funding they provide fulfils a wider societal mission, whilst at the same time providing the highest level of health impact.

In this era of highly competitive funding, even the demonstration of a credible consortium and an extremely relevant research programme no longer guarantees success. Thus, the consortium should ensure that the reviewers, unlikely to be specialists in the specific field, are able to grasp the value and impact of the scientific programme with a very clear statement of aims and impacts, in addition to the scientific impact the research can contribute to the wider research community. Ensuring the funding agency’s understanding of the scientific value may also assist in minimising any future risk of grant reduction during contract negotiation.

Finally, understanding the funding agency’s methods of working after the project has been awarded is critical. A large administrative effort is required by all parties for the reporting to accurately demonstrate the progress of the project. Internal reporting systems for resources and budget must meet the requirements of the funding agency for funds to be provided. A professional and good working relationship with the scientific officer at the funding agency and an assigned project coordinator within the management team with a high level of expertise in managing these aspects proved to be of high importance.

#### Key considerations: Funding

- Ensure clear demonstration of the medical and scientific value to non-expert funding reviewers
- Ensure that administrative processes and systems are in place and meet the requirements of the funding agency and preferably assign a coordinator
- Ensure a professional well-working relationship with the scientific officer at the funding agency

### Patient recruitment

To understand the challenges faced at the start of the clinical programme, we must understand how the patient organisation, the AKU Society, increased the knowledge and identification of AKU patients.

When the AKU Society was founded in 2003, the charity knew of only four people in the United Kingdom (UK) with AKU. Faced with the need for additional evidence of patient numbers to solidify the research and support the case for funding in the UK, the AKU Society sent 11,151 information packs to general practitioners and identified 75 new sufferers [26]. Along with this, it built up a strong presence on emerging social media and forums and began to meet patients in the UK face to face at patient workshops. These workshops served as a crucial way for the new charity to understand the needs and concerns of patients. Once it was realised that a potential treatment for AKU could be nitisinone, but further research was needed to prove the efficacy, the workshops were also used to educate about research and highlight the importance of the upcoming clinical studies with nitisinone.

Armed with this knowledge, and the need to expand the identification of AKU patients across Europe in order to recruit patients into studies, a series of international visits to centres of excellence to speak to local AKU patients and their clinicians and encourage them to consider supporting the studies were performed.

Due to the rarity of AKU, it was soon apparent that it would be necessary to recruit every eligible AKU patient in Europe able to travel to one of the sites. Sixty patients from the UK couldn’t join the study because they were already prescribed nitisinone as an unlicensed medication at the National Alkaptonuria Centre funded by NHS England. This increased the need for international recruitment at the three established study centres. Even patients outside Europe needed to be included to meet the recruitment target, and an expert centre in Jordan supported recruitment through referral of 19 patients. With international recruitment, the coordination was complex when it came to managing the annual visits, including flights and overnight stays, but also in relation to insurance and other administrative matters. Pre-screening was carried out in the patients’ countries to ensure that only eligible patients would be called to the initial screening visit. Translations and interpreters also became important aspects during entire study visits for patients attending a site in a foreign country. In our programme, this was managed through the patient organisation taking ownership of “study participant concierge”-services.

**Fig. 1:**
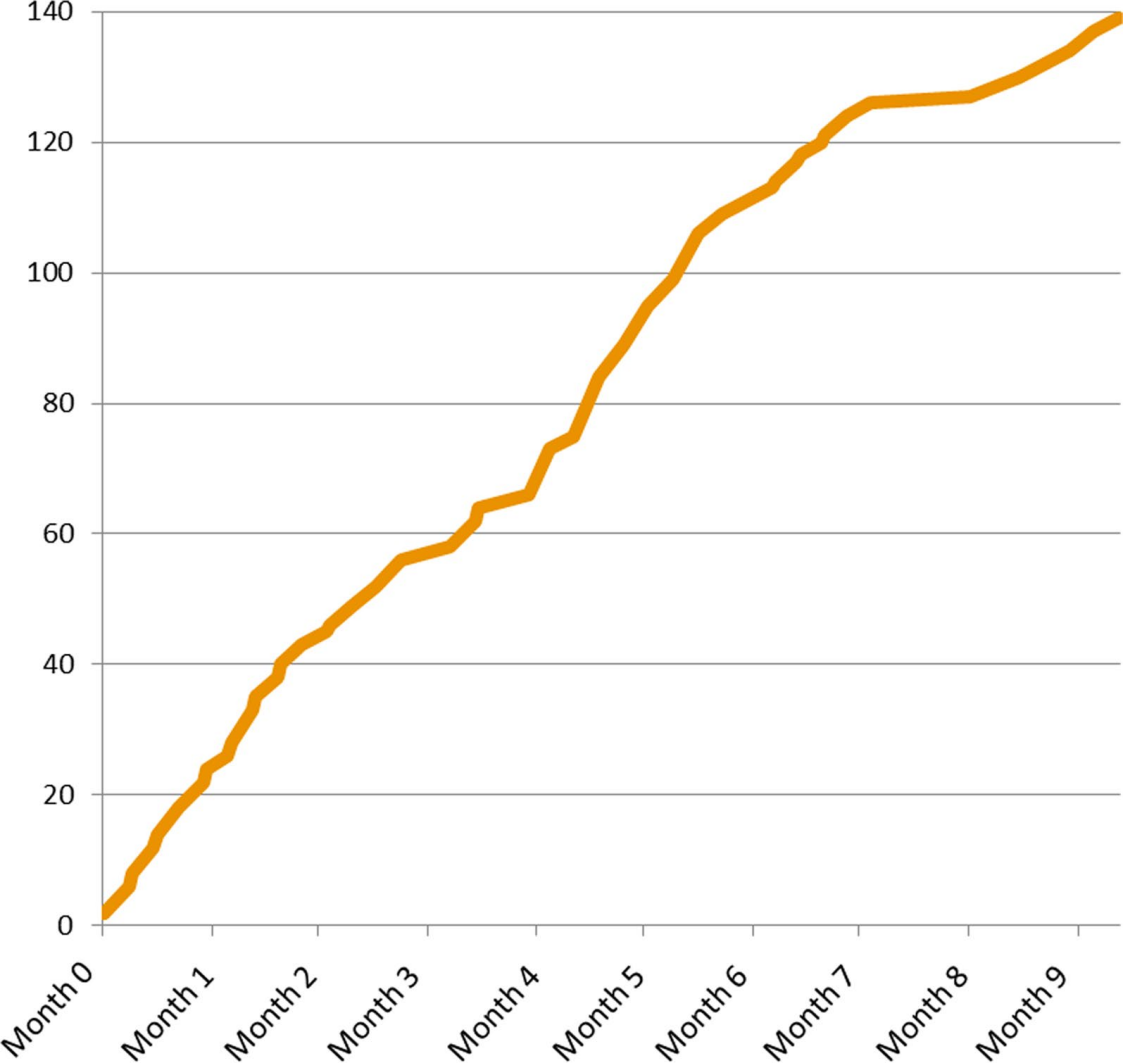
Phase 3 study (SONIA 2) recruitment of 139 patients in a rare disease within 9 months demonstrating a constant recruitment rate throughout the period

The consortium team held weekly recruitment meetings, in addition to the above-mentioned weekly Project Board meetings, during the entire recruitment period to have close management of the progress and resolve any questions or issues. A contributing factor to efficient recruitment (Fig. 1) was that patients were helping by contacting peers outside of the AKU Society’s network. We soon found that patients can be a motivated and vital partner in referring additional patients to the study team. Patients understood the potential benefit of the study for themselves, and for the entire patient community now and in the future. This was shown to them with reference to existing research and by a simple and accessible explanation on the study’s website and via social media. Researchers and doctors across Europe who may have had patients affected by the disease were made aware of the study by Webinar presentations and interactions by the study management team, and a series of email campaigns aimed either directly at them or through membership societies. Informed patients would also often tell their physicians about the clinical study. Due to the relatively low number of patients globally, these doctors are often highly specialised and had other patients with the disease who were informed in turn.

However, there were some problems with patient recruitment at one study centre. AKU patients there had been studied significantly in the past by the medical establishment and were unwilling to participate in what they saw as “yet another study that would not benefit them”. This slowed down recruitment, although eventually resolved through significant interaction between the study site team and the local AKU patients, which highlights the need for communication and open dialogue, taking the concerns of patients into consideration and respecting the experiences of study participants.

### Patient retention

Patients’ needs were also taken into consideration in the design of the studies. This included reducing the amount of time participants spent at the study sites, as the study visits took place over several days and involved long travels. Based on feedback from the project’s initial phase 2 study, the patient organisation highlighted the importance of study participants having ‘down time’ and not staying in hospital as if they were unwell. Due to this, the subsequent phase 3 study hosted participants in nearby hotels. Feedback from this move was overwhelmingly positive. Patients had the opportunity to interact with other patients with the same disease, for many for the first time ever, during the stays. This built a patient community around the studies. Due to the nature of the disease, patients often had severe mobility issues. As a reflection of this, and with feedback from the patient group, it was decided that those participants would be accompanied by a chaperone (usually a family member), who was fully reimbursed. This was a crucial component of patient retention.

Once the recruitment was fully completed, the weekly recruitment meetings within the consortium changed to become retention meetings, handling the close follow-up on patient retention and resolving any potential issues during the entire study period.

We were also faced with the challenge of participants knowing when they were in the no-treatment arm due to the open design. We anticipated that participants would drop out of the study because of not receiving the drug that was potentially beneficial to them. Indeed, one participant resigned from the study after 3 months, realising the medicine could be prescribed off-license in the home country. However, due to the work the AKU Society undertook to highlight the importance of the study and provide educational activities on clinical development and research methodology to the patients from their perspective, this was not widely the case. An AKU patient who enrolled into the non-treatment arm of the study said this:

*‘For me, it is very important to participate in such research because I do not want future generations to go through the same thing as me. I feel it is my duty to contribute to this important research. It is also useful to keep updated on the AKU research developments.*

*‘My view is that the more I can do to help the younger people the better. Professionals need sound evidence on which to base their decisions and challenge people in authority. Hopefully, younger people will benefit as they will be in a position to take nitisinone at an early enough stage to prevent major health issues developing.’*

Ongoing surveying and individual contact by the patient organisations allowed them to track how participants were feeling about all aspects of the studies and triggered ongoing changes in areas that participants were concerned about. For example, the AKU Society took over the task of paying personal and travel expenses and booking flights for participants, based on feedback about delays in reimbursement and issues over flights when this was handled by the study centres. For these patients, who may be disabled to varying degrees, their personal economic situation could be heavily affected by delayed reimbursement for their associated costs. Such aspects of the personal lives of the study participants are important to consider in management of a clinical study. Most participants, when contacted directly, saw this as positive and commended the consortium on the positive impact this had for them.

Having the leading patient organisation within the project team allowed them to be true advocates for the study participants. Any issues highlighted by participants were raised during the next weekly call to the study management team and clinical sites for immediate action. This allowed for the study participation to be as easy as possible for patients, and enabled the high level of retention, which was almost 85%—very high for such a long-term study.

#### Key considerations: Patient recruitment and retention

- Ensure that patient perspective is taken into consideration in the design of the clinical study for optimal patient recruitment and retention
- Ensure close monitoring and management by cross-functional team during the entire study with weekly recruitment/retention meetings
- Ensure timely management of administrative matters for study participants
- Establish study participant advocacy role within the study management team to ensure high patient retention
- Build a community around the clinical study for the study participants

### Multi-stakeholder collaborations: academia and healthcare—patients—pharmaceutical industry

Our project clearly demonstrated the value of collaboration between academia and healthcare, patient organisations and the pharmaceutical industry, with the mutual objective of investigating a new therapy. Involvement of the specific expertise in the core management team enabled an efficient, well-designed and well-executed clinical programme. Some specific areas potentially gaining from these collaborations, as identified within our team, are highlighted below.

*Study design and study operational plan.* Within a collaborative project such as ours, we were able to obtain the clinical experts’ and patients’ perspectives to ensure the feasibility of the design and clinically relevant endpoints. The patient input to the operational plan also ensured a successful study execution, with risks of study drop-outs due to a non-feasible study plan from a study participant perspective minimised.

*Patient recruitment, retention, communication and information.* The patient input to the study information sheets and informed consent forms allowed for the correct level of information, with the optimal language, being provided to potential study participants. Further, as mentioned above, the active recruitment by patient organisations allowed for access to potential study participants. It is, however, important to ensure that all those involved in the patient recruitment processes have adequate Good Clinical Practice knowledge, and that the roles and responsibilities within this process are clearly defined.

*Study participant advocacy.* A unique study participant advocacy was enabled by the equal partnerships where the patient organisations, as members of the consortium management team, were able to be the voice of the study participants, allowed for the optimal execution and operational issue resolution during the conduct of the study. As an example, the leading patient organisation, which had direct contact with all study participants, was able to bring any issue raised by the participants directly to the management team during the weekly calls. This guaranteed immediate resolution of any negative experiences or issues raised by the study participants. These could include administrative issues that negatively affected the experience of the participants, or parts of the operational study plan providing negative experiences during study visits. This kind of study participant advocacy, not normally available during clinical studies, ensured the scientific integrity of the study, where drop-outs due to minor administrative or other issues could be avoided with rapid resolution.

*Regulatory authority and product access processes.* During the interactions with regulatory and reimbursement authorities it is important to ensure relevant clinical disease management expertise, and to provide clinically relevant scientific data, to support their review and thereby the possibility of access to medicines with a high medical need. In our collaborative project the clinical experts’ and patients’ input was ensured from early planning to assessing impact and clinical relevance of study outcomes, thereby ensuring these aspects. The clinical relevance of different symptoms, especially from the patient perspective, was also added as a separate collaborative project to collect these insights that would allow valuation of the Phase 3 data by authorities [27].

## Conclusions

In summary, as for all projects and collaborations it is important to have appropriate preparation, to define the collaborations clearly within a mutually agreed contract that also specifies roles and responsibilities, relationships, decision making, dispute handling and communication, in order to minimise risks of conflicts that hinder the objectives of the collaboration during execution. It is important, especially for the larger partner organisations, that the process for developing and agreeing these are supported across the organisations for full understanding of all terms by all parties. Smaller organisations, such as is usually the case with patient organisations, do not have access to the same supportive functions of legal and other departments as the larger ones, and can otherwise be put in a situation of not having their interests covered within the legal documents.

We found that the equal partnership between all parties in our consortium and the establishment of an active Project Board were the key success factor. This created the momentum that allowed for a successful execution within the challenging timelines that we set for our clinical development programme. A momentum that was based on the strong organisational culture within the consortium where all partners had a high engagement, and where all took ownership of completion of the entire programme which was nurtured by the strong leadership and continuous communication across all partners. The significance of the equal partnerships, and building of the ownership and engagement of individuals, could also be seen for functions that were not standing members of the project board team where some delays and lack of urgency was noted. Therefore, it is important to ensure sufficient engagement of all functions crucial to the project by providing them adequate incentives, such as funding or a publication opportunity.

With the external research grant that partly funded this programme the equal partnership was an opportunity that would not have been available with one partner being the funding source. In order to replicate our successful model, the funding aspect is critical. To establish a functional framework for developing medications for rare diseases which incorporates all these elements, as we did, further attention within the community is needed to develop a model where sustainable funding and risk sharing between private and public organisations allow for such a consortium model to also be successful for future programmes.

Another key success factor was the central role of patient organisation representatives within the management team, and their study participants’ advocacy role. The level at which the patient organisation was incorporated within the study management team is, to our knowledge, unique. The role of the patient organisations in future clinical development processes are to us a clear must. The ability to meet the needs of study participants and include their experiences in real-time during the clinical studies, provide opportunities for the successful outcome of these. The accelerated enrolment into the clinical studies enabled by the partnership with patient organisations was also clearly demonstrated and contributed to the shortening of timelines for this programme.

Finally, the partnerships between academia, patient organisations and industry, with an agreed mutual objective, provide synergies through connecting the strengths of the individual parties. Rather than working in isolation, by bringing all expertise to the same table, the quality and timelines are significantly improved. Important in our success was the consortium environment among all partners, collaborating and respecting the individual expertise for the cause of the project over defined entities. Our project also demonstrates the delayed efforts in research within rare diseases where there is limited knowledge of the biology of the disease and thereby lack of available methodology for designing appropriate research programmes. This despite AKU being the first human genetic disease described, already 120 years ago [28] and said to be instrumental in the understanding of human genetics and inheritance. Bringing all stakeholders together connected all diverse skills and allowed for the progress of research and development within AKU. However, such partnerships require each individual party to be able to maintain their independence and act with integrity. Table 2 provides points to consider for each individual partner.

**Table 2:** Points to consider in collaborative projects between pharmaceutical industry, academia, and patient organisations for grant-funded collaborations

**Patient organisations’ points to consider in collaboration with industry and academia**
Need financial resources from funders to allow patient organisation to hire professional staff Lead representative of patient organisation needs to be on the management committee of the clinical study programme Regular (weekly) contact with the consortium is crucial Patient organisation needs to meet regularly with its patients to make sure it understands their needs properly. Also, needs to carry out regular surveys to gather information on patient satisfaction Patient organisation can be faster than other—such as medical institutions—at reimbursement of patient travel expenses, which otherwise can cause problems and increase patient drop-out due to administrative matters
**Academia’s points to consider in collaboration with industry and patient organisations**
Dialogue between academia and patient groups before, during and after the study programme enables projects Close and real co-operative links between academia and patient groups empower partnership to make the maximum use of skills Academia will not necessarily have the clinical study skills and a good relationship with the pharmaceutical partner is essential for success In addition to contributing effectively to the scientific aspects of the project, pharmaceutical partners bring irreplaceable regulatory expertise needed for successful conclusion of the project Recognition and devolution of activities better delivered by the most appropriate partner allows smooth project flow and less obstacles to successful completion
**Pharmaceutical companies’ points to consider in collaboration with patient organisations and academia**
Ensure adherence to international and national laws and regulations on industry engagement with patient organisations and healthcare professionals Consider ethical aspects in patient recruitment and handling of privacy and security of any personal information when patient organisations are involved in recruitment activities Understand and respect integrity, credibility and independence of the patient organisation and researchers Understand and respect the patient organisations’ and researchers’ working environment and constraints Transparently disclose cooperation Ensure formalised contracting, with clear expectations and roles and responsibilities, prior to start of collaboration

## Data Availability

Data sharing is not applicable to this article as no datasets were generated or analysed during the current study.

## Abbreviations

AKU: Alkaptonuria
ALCAP: Association pour la Lutte Contre L’Alcaptonurie (AKU Society France)
FP7: European Commission’s 7th Framework Programme
EMA: European Medicines Agency
HT-1: Hereditary tyrosinaemia type 1
HGA: Homogentisic acid
RLUH: Royal Liverpool University Hospital
UK: United Kingdom
